# Exploring the mediating role of social support in sports participation and academic burnout among adolescent students in China

**DOI:** 10.3389/fpsyg.2025.1591460

**Published:** 2025-05-19

**Authors:** Jia Gao, Xiaodong Tian, Hailong Wu

**Affiliations:** ^1^School of Physical Education and Health, Zhaoqing University, Zhaoqing, China; ^2^Department of Humanities and Arts, Linyi Vocational University of Science and Technology, Linyi, China; ^3^Department of Exercise and Health, College of Great Health, Tongren University, Tongren, China

**Keywords:** sports participation, academic burnout, social support, adolescent students, intermediary role

## Abstract

**Introduction:**

Academic burnout among adolescent students has been a hot topic of concern among scholars. With the increase of academic pressure, the problem of academic burnout among adolescent students has become more and more prominent, which has a profound impact on their psychological health and academic performance. However, the relationship between sports participation and academic burnout among Chinese adolescent students and its underlying mechanisms remain to be investigated.

**Methods:**

The present study aimed to investigate the relationship between sports participation and academic burnout among adolescent students and to verify the mediating role of social support between the two. In addition, this study used a non-probability convenience sampling technique to investigate the current status of sports participation, academic burnout, and social support among 1,267 Chinese adolescent students.

**Results:**

The results showed that male students scored slightly higher than female students in the sports participation and academic burnout tests, and slightly lower than female students in all social support tests. There was a negative correlation between sports participation and academic burnout among adolescent students (*r* = −0.62). There was a significant negative predictive effect between sports participation and academic burnout among adolescent students (*β* =−0.201, *p* < 0.05) and a positive predictive effect with social support (*β* = 0.231, *p* < 0.05). Social support had a negative predictive effect with academic burnout in adolescent students (*β* =−0.179, *p* < 0.05). Social support had a signiffcant mediating effect between sports participation and academic burnout among adolescent students (*β* =−0.349, *p* < 0.05).

**Conclusion:**

The results of this study provide valuable insight into educational interventions to mitigate and prevent academic burnout among adolescent students.

## 1 Introduction

Academic burnout, as a psychological syndrome characterized by chronic fatigue, a sense of learning detachment, and a diminished sense of achievement, has become a current research hotspot in the field of educational psychology, especially in the context of China, where competition in education is increasing, and the problem is particularly prominent in the adolescent student population (Yuan, 2023; Wu et al., 2023). Studies have shown that academic burnout not only significantly reduces students’ learning efficiency and mental health, but may also predict the onset of depression, posing a serious threat to students’ physical and mental development (Widlund et al., 2023; Flotskaya et al., 2019). Adolescence, as a critical transition period between childhood and adulthood, is an important stage for rapid physical and mental development and the formation of self-identity, but in the face of multiple pressures and expectations from society, school, and family, students are highly susceptible to psychological problems, and academic burnout is one of them (Wang and Fan, 2023; Yuan, 2023). Although scholars at home and abroad have extensively explored the concept, influencing factors, and intervention strategies of academic burnout, most of the existing studies have focused on its negative effects (Chen and Gong, 2022; Gungor, 2019; Tian et al., 2023), and the attention paid to preventive and interventional measures remains insufficient, especially the role of sports participation and social support in alleviating academic burnout has not been adequately studied. In view of the complexity of the psychological state of adolescent students, an in-depth exploration of the specific influencing factors of academic burnout and its mechanisms is not only of great practical significance for the prevention of academic burnout and the promotion of students’ healthy growth, but also has a far-reaching impact on their future careers.

In addition, sports participation, as a positive lifestyle, has received extensive attention in the field of adolescent mental health in recent years, which not only enhances physical fitness, but also significantly improves psychological status and reduces anxiety and depression levels (Herbert, 2022; Bai et al., 2022). In China, although the awareness of adolescents’ sports participation is gradually increasing, their actual participation rate is still low due to excessive academic burden and insufficient sports resources (Zhang et al., 2022). Research suggests that there is a complex association between sports participation and academic burnout, and that moderate sports participation may reduce the incidence of academic burnout by relieving stress and enhancing self-efficacy (Fu et al., 2023). However, despite the widely recognized importance of sports participation, its implementation still faces many challenges, such as insufficient exercise time, single form, and lack of interest, which to a certain extent undermines its role in promoting adolescents’ physical and mental health (Xiong, 2020). The mechanisms of how sports participation specifically affects academic burnout, especially the mediating role of social support in it, still need to be further explored.

Furthermore, studies have shown that social support, as an important external resource for individuals to cope with stress, plays a key role in alleviating academic burnout and promoting physical and mental health, and that good social support can enhance an individual’s psychological resilience, reduce the feeling of stress, and thus improve his or her mental health (Liu and Cao, 2022; Ungar and Theron, 2020). Social support also plays an important role in the relationship between sports participation and academic burnout. On the one hand, social support from families, peers, and teachers can promote students’ active participation in sports and increase their interest and participation in sports; on the other hand, social support can help students maintain a positive mindset in the face of academic pressures, which can indirectly reduce the incidence of academic burnout (Cho et al., 2023). Although existing studies have initially revealed the positive effects of sports participation on academic burnout, there is still a lack of in-depth exploration regarding the mediating role of social support between the two.

In summary, the present study focused on the effects of sports participation on academic burnout among Chinese adolescent students and explored the mediating role of social support. Based on the latest research results at home and abroad and the actual situation of Chinese adolescent students, the study aims to provide new perspectives and scientific basis for alleviating the problem of academic burnout, and at the same time, propose practical guidance for promoting the development of adolescent mental health.

## 2 Literature review and research hypotheses

### 2.1 Sports participation and academic burnout

Academic burnout, which refers to students’ emotional exhaustion, decreased interest in learning, and detachment from school activities due to long-term academic stress, has become an important issue affecting adolescent students’ psychological health and academic performance (Tian et al., 2023; Rehman et al., 2024). Traditional interventions for academic burnout have focused on stress management and curricular adjustments, but recent studies have gradually focused on the potential of exercise participation in alleviating academic burnout (Yu et al., 2022; Chan and Wong, 2024). Exercise participation, as a positive health behavior, not only improves an individual’s physical health, but also significantly reduces stress and improves mood states (Hossain et al., 2024). Students who participate in regular physical activity typically exhibit lower levels of anxiety and depression, and they show greater resilience and positive emotions in the face of academic stress (Kayani et al., 2021; Li et al., 2021). In addition, it has been noted that sports participation can also enhance an individual’s self-efficacy and psychological resilience, thus helping students to better cope with academic stress (Husain et al., 2024). Thus, sports participation may be an important way to alleviate academic burnout. Despite the abundance of research on sports participation and mental health, the relationship between sports participation and academic burnout has not been fully explored, especially in adolescent student populations. It can be inferred that there may be a high negative correlation between sports participation and academic burnout. Therefore, Hypothesis 1 of this study is proposed: sport participation and academic burnout are negatively correlated.

### 2.2 Mediating role of social support

The mediating mechanism in this study is the mediating role of social support. Research has shown that social support plays an important role in alleviating academic burnout (Wang et al., 2021). Social support includes emotional, informational, and instrumental support from peers, families, and teachers, which can help students better cope with academic stress and enhance their sense of belonging and psychological resilience (Jolly et al., 2021). A study found that students with higher levels of social support had significantly lower levels of academic burnout, and social support can also be used to provide emotional comfort and practical help, which can enhance students’ coping skills and reduce the psychological burden caused by academic stress (Shankland et al., 2019). It can be seen that there is a significant negative correlation between social support and academic burnout. Second, scholars point out that sports participation is not only beneficial to students’ physical health, but also closely related to social support. Because, sports activities usually involve teamwork and social interactions, students who participate in sports activities tend to receive more social support (providing opportunities to build positive relationships with peers, teachers, and family members), which also enhances students’ sense of belonging and social connectedness, which further enhances the quality of social support (Wilson, 2021). Therefore, it can be inferred that sport participation not only directly improves students’ mental health, but also indirectly plays a positive role by enhancing social support. Finally, scholars have also noted that students who participate in sports activities are able to cope with academic stress more effectively through greater social support, thus reducing levels of academic burnout (Baria and Gomez, 2022). It can be inferred that social support may be an important mediating mechanism for sports participation to influence academic burnout. Therefore, Hypothesis 2: Social support is negatively related to academic burnout; Hypothesis 3: Exercise participation and social support are positively related; and Hypothesis 4: Social support mediates the relationship between exercise participation and academic burnout among Chinese adolescent students are proposed, respectively.

**Figure 1:**
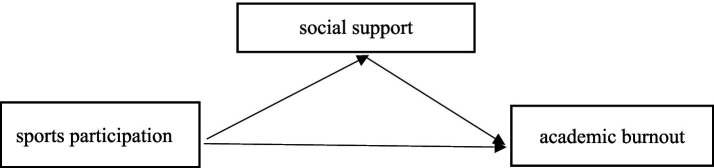
Research framework diagram.

In summary, scholars have pointed out the association between sports participation and Chinese adolescent students’ academic burnout and emphasized that sports participation may indirectly affect adolescent students’ academic burnout through social support, and the above findings provide a theoretical basis for the hypotheses of this study. Therefore, the conceptualization of the research structure of this study (Figure 1) was established: (1) to test the predictive role of sports participation and academic burnout among Chinese adolescent students; (2) to examine the mediating role of social support between sports participation and academic burnout among Chinese adolescent students.

## 3 Research objects and methods

### 3.1 Research subjects

This study used a non-probability convenience sampling technique to measure 1,267 adolescent students in Guangzhou City, China. From March 2024 to May 2024, 2 schools were selected from 11 precincts (Yuexiu, Liwan, Haizhu, Tianhe, Baiyun, Huangpu, Panyu, Huadu, Nansha, Zengcheng, Conghua) in Guangzhou City (22 schools in total). Subjects were tested in the physical education classroom, with the primary testers being professionally trained physical education teachers and psychology students. The tests were approved by the leaders of the testing schools, the physical education teachers, and the subjects and their parents, and all questionnaires were completed within 10 min (paper questionnaires were distributed on site). The questionnaires were administered in a group setting, and the subjects were instructed to complete the questionnaires using standardized instructions. The inclusion criteria for subjects prior to the administration of the questionnaires were: (1) Chinese students in the adolescent stage; (2) those who were able to participate in physical activity on their own. Subjects who would not meet the above criteria would be excluded from the study. After the questionnaires were retrieved, exclusion was made according to the following criteria: (1) non-pubertal students; (2) people with disabilities; and (3) missing data. In this study, 1,300 questionnaires were distributed, the total number of questionnaires recovered was 1,283, and the number of validly recovered questionnaires was 1,267, with an effective recovery rate of 97.46%. The average age of the respondents was 13.5 years old, with a balanced ratio of male and female students (697 boys and 570 girls). Among them, the sample size calculation formula used in this study is: N = Z^2^ × (P × (1-P)/E^2^) (Suresh and Chandrashekara, 2012), the sample size calculated by the formula is 1,165, in order to avoid the emergence of data that are sufficient to represent the whole due to factors such as missing data, it is formulated in the calculation of 1,165 to issue the questionnaires upgraded to 1,300 in order to ensure the representativeness of the questionnaire data. In addition, in order to ensure the reliability of the data in this study, anonymity, standardized instructions, and supervision of questionnaire distribution were adopted in accordance with the Declaration of Helsinki. The study was approved by the Ethics Committee of Zhaoqing University (No. 2024025), and all subjects and their parents/guardians signed an informed consent form. The questionnaires for this study were tested and collected from October 20, 2024 to November 25, 2024.

## 4 Research methods

### 4.1 Measurements

#### 4.1.1 Exercise participation scale

The Physical Activity Rating Scale (PARS-3) developed by Liang (1994) was used to assess the exercise participation level of secondary school students, mainly in terms of intensity, duration and frequency of exercise. The scale consists of four questions, the first three of which relate to exercise intensity (how intensively do you perform physical activity?) The first three questions relate to the intensity (how hard do you do it?), duration (how many minutes do you do it for?), and frequency (how many times a month do you do it?) and frequency (how many times a month do you do the above physical activity?). Each question had one to five options and was scored on a scale of one to five. The fourth question was an open-ended question about the participant’s preferences (What kind of physical activity do you like?). The total physical activity score ranged from 0 to 100 and was calculated as Exercise = Exercise Intensity * Exercise Time * Exercise Frequency. According to the total score of the scale, physical activity can be categorized as follows: less than 19 indicates little exercise, 20 to 42 indicates moderate exercise, and more than 43 indicates a lot of exercise. And in previous studies in related fields involving adolescent students, the Exercise Participation Scale was used to measure the current status of exercise participation among adolescent students with good reliability and validity (Zhao and Xie, 2023). In this study, the Cronbach’s alpha coefficient of the scale was 0.63, and the KMO value was 0.76, indicating that the scale has high internal consistency and good reliability and validity.

#### 4.1.2 Adolescent academic burnout scale

The Adolescent Academic Burnout Scale, developed by Yan Wu and Xiaoyang Dai, was used to reflect students’ sense of personal achievement in learning (Wu et al., 2007; Wu et al., 2010). The scale consists of 16 items, including three sub-dimensions: physical and mental exhaustion (e.g., topic 2, I feel empty in my heart lately, I do not know what to do), academic detachment (e.g., topic 3, I’m so bad at my studies, I really want to give up.) and low achievement (e.g., Question 15, I always manage to cope with academic problems easily). Each item of the scale was rated on a 5-point Likert scale, i.e., 5 for “fits very well,” 4 for “fits somewhat,” 3 for “not quite sure,” and 3 for “not quite sure. “Not quite” is scored as 2, and ‘Not at all’ is scored as 1. The scale uses summation to calculate the total score, and the higher the score, the more serious the degree of academic burnout. In previous research in the field of adolescent students, the Adolescent Academic Burnout Scale has been used to measure the current status of adolescent academic burnout among adolescent students, and it has good reliability and validity (He et al., 2022). In this study, the Cronbach’s alpha coefficient of the scale was 0.71, and the KMO value was 0.82, which indicated that the internal consistency of the scale was high and had good reliability and validity.

#### 4.1.3 Social support scale

This scale was developed by Zimet et al. (1988) and later translated and revised by the Chinese scholar Jiang (2001) to reflect the degree of individual’s understanding of social support, emphasizing the individual’s self-understanding and self-perception of different sources of social support (Jiang, 2001). The scale is divided into 12 questions and consists of three dimensions: family support (e.g., Question 3, My family can help me in a concrete way), friend support (e.g., Question 6, My friends can really help me), and teacher support (e.g., Question 1, My teacher is there for me when I have problems). The scale is scored on a 7-point Likert scale from 1 to 7, ranging from “Strongly Disagree” to “Strongly Agree,” and the total score is calculated by summing the scores, with total scores ranging from 12 to 36 for low support, 37–60 for intermediate support, and 61–84 for intermediate support. A total score of 12–36 is considered low support, a total score of 37–60 is considered intermediate support, and a total score of 61–84 is considered high support, with higher scores indicating higher levels of perceived social support (Jiang, 2001; Ma et al., 2024). In previous studies in related fields involving adolescent students, the Social Support Scale was used to measure the status of social support among adolescent students with good reliability and validity (Jiang et al., 2024). In this study, the Cronbach’s alpha coefficient of the scale was 0.86 and the KMO value was 0.88, indicating that the scale has high internal consistency and good reliability and validity.

### 4.2 Mathematical statistics

In this study, data were analyzed using SPSS 26.0 and the PROCESS plug-in to explore the relationship between exercise participation, academic burnout and social support, and to test the mediating role of social support between exercise participation and academic burnout. First, sample data on Chinese adolescent students’ sports participation, academic burnout, and social support were collected by questionnaire survey method, and the data were preprocessed, including missing value treatment, outlier detection, and normality test. Next, descriptive statistics were used to analyze the basic characteristics of the sample data, and independent samples t-test and one-way ANOVA were used to explore the differences between gender and age on each variable. Subsequently, Pearson’s correlation analysis was used to investigate the correlation between athletic participation, academic burnout, and social support, and mediation effect tests were conducted through Model 4 of the PROCESS plug-in, setting athletic participation as the independent variable, academic burnout as the dependent variable, and social support as the mediator, and using 5,000 Bootstrap samples to assess the significance of the direct effect, indirect effect, and total effect. In this paper, *p* < 0.05 was set as the statistical result of data analysis and significant.

## 5 Results

### 5.1 Descriptive statistics of the current status of sports participation, social support, and academic burnout

The statistical results in Table 1 show that athletic participation, social support, and academic burnout were statistically significant when analyzing gender differences (*p* < 0.05); athletic participation and academic burnout were statistically significant when analyzing age differences (*p* < 0.05), while social support was not statistically significant. Male students scored slightly higher than female students in the sports participation and academic burnout tests, while they all scored slightly lower than female students in the social support test. In addition, the three variables showed some regularity in different statistical calibers, which helps to further understand the degree of influence and interrelationship between sports participation, social support, and academic burnout.

**Table 1:** Descriptive statistics of test results for sports participation, social support, and academic burnout (*M* ± *SD*).

Sex	*N*/people	Sports participation	Social support	Academic burnout
Male	697	46.30 ± 3.43	37.12 ± 2.51	48.23 ± 8.22
Female	570	45.98 ± 6.21	37.11 ± 2.65	45.26 ± 9.25
Total	1,267	46.14 ± 4.82	37.12 ± 2.53	46.75 ± 8.74
Gender difference (*F/p*)		2.45/0.03^*^	3.18/0.00^*^	4.22/0.00^*^
Age difference (*T/p*)		3.78/0.00^*^	0.70/0.65	2.72/0.00^*^

*N* = 1,267; *p < 0.05.

### 5.2 Common method bias test

To assess the possibility of common method bias, a Harman one-way test was conducted. The test consisted of an exploratory factor analysis where all measured variables were loaded onto a single factor to determine the extent to which common method variance existed. The results showed that the first factor accounted for 32.43% of the total variance, which is below the 40% threshold (Cooper et al., 2020). This indicates that common method bias was not a significant issue in this study, which affirms the validity of the data in this study and increases the credibility of the findings.

### 5.3 Correlation analysis

The results of the correlation analysis showed (see Table 2) that sports participation and academic burnout were significantly positively correlated with social support, and social support was significantly negatively correlated with academic burnout, which verified the initial hypothesis of this study and provided a better foundation for the subsequent mediation effect test of this study.

**Table 2:** Correlation analysis statistics between sports participation, social support, and academic burnout.

Variant	*M* ± *SD*	Sports participation	Social support	Academic burnout
Sports participation	46.14 ± 4.82	1		
Social support	37.12 ± 2.53	0.67^**^	1	
Academic burnout	46.75 ± 8.74	−0.62^*^	−0.71^**^	1

*N* = 1,267; *p < 0.05, **p < 0.01.

### 5.4 Tests of mediating effects of athletic participation and academic burnout

Using athletic participation as the independent variable, social support as the mediator variable, and academic burnout as the dependent variable, the SPSS macro plug-in PROCESS provided by Hayes (2022) was used to select Model 4 based on Templates, and a bias-corrected, non-parametric percentile Bootstrap test (5,000 repetitive samples) was selected to calculate the 95% confidence intervals for mediation effects analysis. After controlling for gender and age, the results showed a negative correlation between athletic participation and academic burnout, *β* = −0.201, *p* < 0.05, supporting hypothesis 1. Social support was negatively correlated with academic burnout, *β* = −0.179, *p* < 0.05, supporting hypothesis 2. Athletic participation was positively correlated with social support, *β* = 0.231, *p* < 0.05, supporting hypothesis 3. Social support played a role in the relationship between athletic participation and academic burnout mediates the relationship, *β* = −0.349, *p* < 0.05, supporting hypothesis 4 (Table 3).

**Table 3:** Regression analysis of the mediation model between sports participation and academic burnout.

Variant	Social support		Academic burnout	
	*β*	*t*	*β*	*t*
Age	−0.128	−3.923*	−0.098	−3.081*
Sex	−0.121	−3.131**	−0.083	−2.956**
Sports participation	0.231	5.582**	−0.201	−4.568*
Social support			−0.179	−4.128*
*R* ^2^	0.153		0.136	
*F*	123.31		103.75	

*P < 0.05, **P < 0.01.

**Figure 2:**
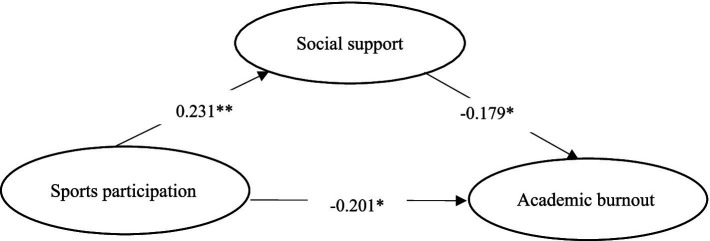
Chain mediation model diagram of social support between athletic participation and academic burnout.

Table 4, Figure 2 shows the results of the mediation effects test with Bootstrap 95% confidence intervals for the indirect effect of athletic participation on academic burnout excluding 0. These results further support Hypotheses 1 through 4 (Figure 2).

**Table 4:** Test of the mediating effect of social support in sports participation and academic burnout.

Type of effect	Efficiency value	Boot SE	Bootstrap95% CI		Effect proportion
			Lower limit	Limit	
Total effect	−0.349	0.031	−0.062	−0.018	100%
Sports participation → academic burnout	−0.166	0.015	−0.031	−0.025	47.56%
sports participation → social support → academic burnout	−0.183	0.026	−0.073	−0.031	52.44%

## 6 Discussion

### 6.1 Sports participation and academic burnout

This study found that sports participation was significantly negatively correlated with academic burnout among Chinese adolescent students, a result that is consistent with the findings of previous research: students who participated in sports activities scored significantly lower than those who did not participate in sports activities on the core dimensions of academic burnout (emotional exhaustion, decreased interest in learning, and feelings of detachment from school activities) (Zhang, 2023). The reason for this is that, firstly, scholars believe that physical activity can effectively reduce the level of stress, which is one of the main triggers of academic burnout. This is because, according to the stress relief theory, physical exercise can act as a mood regulator by stimulating the secretion of endorphins, thus relieving individuals’ negative emotions and stress perceptions (Hossain et al., 2024). Studies have shown that adolescents who regularly participate in physical activity have significantly lower levels of perceived stress than their non-exercising peers, and that lower levels of stress are closely related to a reduction in academic burnout (Wang, 2023; Fu et al., 2023). In addition, physical activity can further alleviate academic burnout by improving sleep quality. Studies have shown that students who regularly engage in physical activity on a weekly basis have significantly higher sleep quality scores than those who do not engage in physical activity, and these students have significantly lower scores on the emotional exhaustion dimension of academic burnout (Zhu et al., 2021). These findings are consistent with the findings of this study and further validate the important role of sport participation in alleviating academic burnout. Second, recovery theory also suggests that physical activity can provide individuals with a break from academic tasks, thereby helping students to rejuvenate, alleviate fatigue, and regain motivation to learn (Teuber et al., 2024). One study noted that students who participated in extracurricular sports activities tended to exhibit lower academic stress and higher motivation, students who participated in team sports scored significantly higher on the self-efficacy measure than those who did not, and increased self-efficacy was effective in buffering against academic burnout, such as low achievement and decreased interest in learning (Yang et al., 2022; Van Raalte and Posteher, 2019). As can be seen, the above findings are consistent with the results of the present study, further illustrating the multifaceted benefits of exercise participation in alleviating academic burnout. These studies emphasize the importance of integrating regular physical activity into the daily lives of secondary school students as a preventive measure against academic burnout.

Furthermore, the results of the data research show that there are gender differences in athletic participation, social support, and academic burnout, and there are age differences in athletic participation and academic burnout. Male students scored slightly higher than female students in the sports participation and academic burnout tests, while they scored slightly lower than female students in the social support test. This is consistent with the results of previous studies. The reasons for this are, firstly, boys scored significantly higher than girls on sports participation and academic burnout, which may be related to gender role socialization. This may be related to the socialization of gender roles, because in traditional sociocultural contexts, boys are usually encouraged to participate in more competitive and physically demanding sports activities, whereas girls may be directed to participate in more moderate or non-competitive activities (Spallacci, 2020; Roselló-Novella et al., 2023). In addition, male students are more likely to use problem-oriented coping strategies in the face of academic stress, whereas female students are more likely to use emotion-oriented coping strategies (Kahvazi, 2022). Therefore, this difference may lead to higher scores on the emotional exhaustion dimension of academic burnout among male students. Secondly, girls scored significantly higher than boys on social support, which may be related to gender differences in social behavior and emotional expression. This is because research has shown that girls are more inclined to establish close social relationships and are more proactive in seeking and providing emotional support (Archer, 2019). This social tendency makes girls score higher on the emotional and instrumental support dimensions of social support.

In addition, there is a significant difference between sports participation and academic burnout in terms of age, which may be related to the physiological and psychological developmental characteristics of adolescent students. As students grow older, academic stress gradually increases, especially during the promotion stage (e.g., middle school to high school), which may lead to a significant increase in the level of academic burnout (Kim, 2020). Older students may face more academic tasks and higher expectations, which further exacerbates the level of academic burnout. On the other hand, older students’ lower scores on athletic participation may be related to the increased academic load and adjustments in time allocation. As their academic load increases, students may spend less time engaging in physical activities and instead devote more energy to their studies, so changes in time allocation may lead to lower levels of sports participation (Van Dyck et al., 2015). It can be seen that the existence of gender and age differences reflect the influence of physical, psychological and socio-cultural factors on sports participation, social support and academic burnout among adolescent students, and the above differences provide an important basis for developing targeted intervention strategies.

### 6.2 Mediating role of social support

This study found that social support mediates the relationship between sports participation and academic burnout among Chinese adolescent students, a finding that is consistent with the findings of previous studies that social support is negatively related to academic burnout. First, social support, as an important protective factor, can help students alleviate academic stress and reduce the incidence of academic burnout by providing emotional support, instrumental help, and information resources (Romano et al., 2020; Liu and Cao, 2022). Scholars point out that support from families can reduce students’ loneliness and exhaustion due to academic stress by providing emotional comfort and practical help. Support from peers and teachers, on the other hand, can help students better adapt to academic challenges by enhancing the sense of belonging and recognition (Cho et al., 2023). Another study also showed that students with higher levels of social support scored significantly lower on the emotional exhaustion dimension of academic burnout than those with lower levels of social support, suggesting that social support is effective in buffering the negative effects of academic stress on students’ mental health (Ye et al., 2021; Popa-Velea et al., 2017). The above findings further validate the important role of social support in alleviating academic burnout.

In addition, the present study found a significant positive relationship between sport participation and social support, a result that is consistent with the findings of previous studies. Sports participation, especially team sports and group exercise, provides students with more opportunities for social interaction and can help them establish and maintain positive social relationships, which can enhance a sense of belonging and well-being (Lee et al., 2023). An empirical study found that students who regularly participated in team sports on a weekly basis had significantly higher social support scores than those who did not, further suggesting that sports participation promotes social interaction and teamwork, which in turn enhances students’ social support (Golaszewski et al., 2022). Finally, social-ecological system theory suggests that individuals’ behaviors and psychological states are influenced by multi-level social environments, and that social support, as an important social resource, can mitigate the negative effects of academic burnout by enhancing individuals’ psychological resilience and sense of belonging (Yu et al., 2024). Whereas students who participated in sports activities were able to cope with academic stress more effectively by obtaining more social support, thus reducing the incidence of academic burnout, students who lacked social support still had higher levels of academic burnout even though they participated in sports activities, which is sufficient to show that social support plays an important role in the relationship between sports participation and academic burnout (Ye et al., 2021; Ji, 2024). It can be seen that the above findings are consistent with the present study, further validating the mediating role of social support in the relationship between athletic participation and academic burnout. In conclusion, the results of the present study emphasize that while promoting sports participation, attention should be paid to enhancing students’ social support levels to more effectively alleviate academic burnout and provide comprehensive intervention strategies for adolescent students’ mental health and academic development.

### 6.3 Implications

By exploring the relationship between exercise participation, social support and academic burnout among Chinese adolescent students, this study reveals the mediating role of social support between exercise participation and academic burnout, and provides a new theoretical perspective and practical basis for understanding the mechanism of academic burnout alleviation. The findings suggest that exercise participation not only can alleviate academic burnout by directly reducing stress levels and improving emotional states, but also can indirectly play a positive role by enhancing social support networks. From the practical level, this study emphasizes the importance of incorporating regular physical activity into the daily education system of secondary school students, while pointing out the critical role of building a multidimensional social support network in alleviating academic burnout.

The findings of this study can be interpreted within the cultural context of China, where academic achievement is highly emphasized and often prioritized over extracurricular activities such as sports (Zuo et al., 2023). In such a high-pressure educational environment, social support—particularly from parents and teachers—may play a more pronounced role in mitigating academic burnout and encouraging sports participation (Wills, 1985). In addition, the collectivist orientation of Chinese culture, which values interdependence and group harmony, may also amplify the impact of social support, making it a more salient protective factor compared to more individualistic societies. Therefore, while the mediating role of social support identified in this study offers valuable insights, its strength and nature may differ in other sociocultural contexts (Glazer, 2006). Future research is encouraged to replicate this model in different cultural settings to assess its generalizability and to explore how cultural norms shape the interplay between sports, support systems, and academic well-being.

In addition, drawing on the social-ecological system theory, the current findings not only deepen our theoretical understanding of the mechanisms linking sports participation, social support, and academic burnout, but also provide a multilevel framework for guiding effective interventions. At the individual level, educators and policymakers can design personalized physical activity programs that align with students’ interests and developmental needs, thereby enhancing motivation and sustained participation. At the family level, parent education programs can be implemented to strengthen emotional and instrumental support for adolescents, particularly by encouraging parental involvement in their children’s leisure and academic activities. At the school level, schools can foster a supportive climate by integrating structured physical education into the curriculum, providing access to diverse sports facilities, and building peer-support networks through group-based exercise initiatives.

### 6.4 Limitation and future research directions

While the present study provides valuable insights into the complex interactions between athletic participation, social support, and academic burnout, there are some notable shortcomings and directions for future research. First, the cross-sectional design of this study limited its ability to establish causal relationships or infer temporal relationships between variables. Future research should employ longitudinal or experimental designs to more rigorously examine the causal mechanisms underlying these relationships and to capture potential changes over time. Second, this study focused primarily on the middle school student population, and the generalizability of the findings may be limited. Future research should consider expanding the sample to include participants from diverse regions—particularly those with varying levels of economic development and cultural backgrounds—as well as different educational stages (e.g., primary school, high school, or university students). Third, the current study only considered a mediation model, which may not fully capture the complexity of the relationships among the studied variables. Future research could benefit from exploring alternative theoretical models, such as moderated mediation or moderated models more broadly, to examine whether individual differences (e.g., personality traits, coping strategies) or contextual factors (e.g., school environment) might condition the strength or direction of these associations. Lastly, this study relied primarily on self-reported data, which may be subject to biases such as social desirability and recall errors. To enhance the reliability and validity of future findings, subsequent research could incorporate multiple data sources (e.g., teacher assessments, parent reports, or objective behavioral indicators) and adopt mixed-methods approaches. Addressing these methodological limitations will contribute to a more nuanced understanding of the complex and dynamic interplay among sports participation, social support, and academic burnout, thereby providing a stronger empirical foundation for the development of targeted and evidence-based intervention strategies.

## 7 Conclusion

This study found that sports participation and academic burnout were negatively correlated, social support was also negatively correlated with academic burnout, and sports participation was positively correlated with social support. Social support mediated the relationship between sports participation and academic burnout. The study emphasizes that physical activity and social support networks are essential to alleviate academic burnout. However, there are limitations in the design of this study, such as a cross-sectional design that restricts causal inference and limited sample generalizability. Future studies need to adopt a longitudinal design, expand the sample size, incorporate moderating variables and use multiple sources of data for analysis to deepen understanding and enhance the reliability of the study.

### 7.1 Recommendations

In response to the findings of this study, the following three recommendations are made to address academic burnout, sports participation, social support, and gender differences among Chinese adolescent students:

(1) Strengthen physical education and promote gender-equitable sports participation.

Schools should pay attention to the role of physical education in alleviating academic burnout, and encourage equal participation of boys and girls in sports activities by optimizing curriculum and activity design. For example, to address the problem of lower sports participation among girls, more sports programs (such as yoga, dance, etc.) can be designed to suit girls’ interests and needs, while gender stereotypes can be eliminated through publicity and education to encourage girls to participate in more competitive and physically demanding sports. For boys, diversified forms of sports and psychological counseling can be used to help them release pressure and improve their mental health in sports.

(2) Building a multi-level social support network.

Schools, families and society should work together to provide students with multi-dimensional social support. Schools can help students get more emotional support and academic guidance by establishing student support groups, strengthening teacher-student interactions, and conducting mental health education. At the family level, parents should pay attention to their children’s psychological state, provide emotional support and practical help, and create a warm family atmosphere. In addition, the social level can provide more supportive resources for students through community activities or public welfare programs, especially for the problem of low social support for male students, encouraging them to take the initiative to seek help and improve the utilization rate of social support.

(3) Develop targeted intervention strategies to alleviate academic burnout.

Personalized intervention strategies are developed to alleviate academic burnout according to the characteristics of students of different genders and age groups. For male students, psychological counseling and time management training can be provided to help them cope with academic stress more effectively; for female students, the risk of academic burnout can be further reduced by strengthening the social support network. In addition, schools should reasonably adjust the academic load, especially for older students, and provide more time and opportunities to participate in sports activities to help them release pressure and enhance their mental toughness in sports, so as to achieve a balance between academics and physical and mental health.

## Data Availability

The raw data supporting the conclusions of this article will be made available by the authors, without undue reservation.
