# Supplementary material for: Exploring the mediating role of social support in sports participation and academic burnout among adolescent students in China
Source: Front Psychol. 2025 May 19;16:1591460. doi: 10.3389/fpsyg.2025.1591460 (PMC12127404; doi:10.3389/fpsyg.2025.1591460)
Supplement: Supplementary file 1 [file Data_Sheet_1.zip › scale/Physical Activity Rating Scale (PARS-3).docx]

**Physical Activity Rating Scale (PARS-3)**

The following questions measure how physically active you have been during the past month. For the first three questions, choose the most appropriate one and write the answer number in parentheses after the question, and for the fourth question, underline the program in which you are exercising, or write the program in which you are exercising.

1、What is the intensity of your physical exercise?

① Light exercise (e.g., walking, doing radio gymnastics, playing goalball, etc.)

② small intensity of less intense sports (such as recreational volleyball, table tennis, jogging, tai chi, etc.)

③ moderate intensity of the more intense and long-lasting exercise (such as cycling, running, playing table tennis, etc.)

④Hasty breathing, sweating a lot of high-intensity, but not long-lasting sports (such as playing badminton, basketball, tennis, soccer, etc.)

(5) High-intensity, long-lasting sports that involve a lot of shortness of breath and sweating (e.g., running, aerobics, swimming, etc.).

2、How many minutes do you spend on the above sports activities?

① Less than 10 minutes

②11 to 20 minutes

③21 to 30 minutes

④31 to 59 minutes

⑤ More than 60 minutes

3、How many times a month do you do the above sports activities?

① Less than once a month

②3 to 5 times a week

③2 to 3 times a month

④Approximately 1 time per day

⑤ 1 to 2 times a week

4、What kind of sports do you like?

①walking, running

②Traveling, picnic

③Sports dance

④Ball games

⑤ Rope skipping

⑥ Taijiquan, health techniques

⑦ Fitness equipment activities

⑧ Swimming

⑨ Others

Description:

The amount of exercise was tested using the Physical Activity Rating Scale (PARS-3) revised by Liang Deqing et al. This scale examines the amount of exercise from the intensity, time and frequency of participating in physical activity. Exercise amount=intensity×time×frequency, intensity and frequency from 1 to 5 levels, respectively, 1 to 5 points, time from 1 to 5 levels, respectively, 0 to 4 points, the highest score is 100 points, the lowest score is 0 points, the exercise amount assessment criteria: ≤19 for fluctuation; 20~42 for medium exercise; ≥43 for large exercise. the re-test reliability of the PARS-3 is 0. 82.
