# Supplementary material for: Exploring the mediating role of social support in sports participation and academic burnout among adolescent students in China
Source: Front Psychol. 2025 May 19;16:1591460. doi: 10.3389/fpsyg.2025.1591460 (PMC12127404; doi:10.3389/fpsyg.2025.1591460)
Supplement: Supplementary file 1 [file Data_Sheet_1.zip › scale/Adolescent Burnout Scale.doc]

**Introduction to the Adolescent Burnout Scale**

The concept of learning burnout is derived from job burnout, which is a persistent, negative, learning-related psychological state that occurs in normal people. This state is characterized by: ① energy depletion and emotional exhaustion. ② Enthusiasm for learning-related activities gradually disappears, and negative attitudes toward academics are held. (iii) Individuals do not experience a sense of achievement or efficacy in their studies. Most researchers believe that burnout consists of three dimensions: physical and mental exhaustion, academic detachment, and low achievement. Studies have found a correlation between academic burnout and symptoms such as anxiety and depression.

The Adolescent Learning Burnout Scale, developed by Yan Wu and Xiaoyang Dai in 2007, is based on the Maslach Burnout Questionnaire and assesses adolescents' learning burnout. The scale is based on the Maslach Burnout Questionnaire, which can be used to assess the burnout situation of adolescents.

I. Content and implementation method

The Adolescent Learning Burnout Scale (ALBS) is a self-assessment scale that consists of 3 dimensions, including physical and mental exhaustion, academic detachment, and low achievement, with a total of 16 entries. A five-point scale was used, i.e., “very much in line” was scored as 5 points, “somewhat in line” was scored as 4 points, “not quite sure” was scored as 3 points, “not quite in line” was scored as 2 points, and “not quite in line” was scored as 2 points. 2 points for “not quite” and 1 point for “very much”. Some entries were reverse scored.

Scoring Methods and Applications

Physical and Mental Exhaustion Subscale: includes 4 items of 2, 5, 8, and 12. These items reflect the individual's feelings after learning. As well as the depletion and fatigue condition due to learning.

Academic detachment subscale: includes 3, 6, 9, 10, 13 total 5 items. These items reflect a negative attitude of the individual towards learning.

Low Achievement subscale; includes 7 items totaling 1, 4, 7, 11, 14, 15, and 16. These items reflect an individual's relatively low personal achievement in learning.

The reverse scoring entries are 1, 4, 7, 14, 15, and 16 totaling 6 items. The sum of the scores of all 16 items is the total score of the scale, which reflects the overall status of the participant's academic burnout. Since the scale has just been completed, the actual application effect has yet to be tested in subsequent studies.

II. Test Questions:

Dear students: In order to better understand your situation regarding learning, we sincerely invite you to assist us in completing the following questionnaire. The content of this questionnaire will be treated confidentially, please feel free to fill in truthfully. Please feel free to fill in the questionnaire truthfully. There is no right or wrong answer, and we are looking forward to your truthful answers. Please do not leave anything out. Thank you for your cooperation!

1. Please put a tick in the box that corresponds to your actual situation.

2. Please answer each question without omitting anything or taking time to think about it. If you are not quite sure, please answer after making reasonable assumptions, choosing only one for each question.

Grade ____________ Gender ____________

Items 5 means very much 4 means somewhat not 3 means not quite sure 2 means not quite 1 means very much not

1. I can be energetic and engaged in my studies. 5 4 3 2 1

2. I feel empty lately and don't know what to do. 5 4 3 2 1

3. I am so bad at studying that I want to give up. 5 4 3 2 1

4. I am able to reach my goals on a regular basis.

5 4 3 2 1 5. At the end of a day's study, I feel extremely tired. 5 4 3 2 1

6. I feel like it doesn't matter if I don't know what I'm learning anyway. 5 4 3 2 1

7. When studying, I forget everything around me. 5 4 3 2 1

8. In recent times, I often feel exhausted. 5 4 3 2 1

9. I don't feel a sense of accomplishment when it comes to my studies. 5 4 3 2 1

10. I feel that studying is meaningless to me. 5 4 3 2 1

11. I can cope with exams well. 5 4 3 2 1

12. I often feel exhausted at school. 5 4 3 2 1

13. I study with a cynical attitude. 5 4 3 2 1

14. I can effectively solve problems that arise in my studies. 5 4 3 2 1

15. I am always able to cope with problems in my studies easily. 5 4 3 2 1

16. I can easily grasp what I have learned. 5 4 3 2 1
