# Supplementary material for: Exploring the mediating role of social support in sports participation and academic burnout among adolescent students in China
Source: Front Psychol. 2025 May 19;16:1591460. doi: 10.3389/fpsyg.2025.1591460 (PMC12127404; doi:10.3389/fpsyg.2025.1591460)
Supplement: Supplementary file 1 [file Data_Sheet_1.zip › scale/Collaborative Social Support Scale.doc]

Collaborative Social Support Scale

The Zimet Perceived Social Support Scale introduced by Blumenthal et al. (1987) was modified by Jiang Qianjin. The scale consists of 12 items on a seven-point scale from 1-7, with higher scores indicating a higher level of perceived social support. The internal consistency coefficient α of this scale in this study was 0.93. (three dimensions of family support friends support other support )

**1 for Strongly Disagree，2 for Slightly disagree，3 for Neutral，4 for Slightly agree，5 for	Strongly agree，6 for Strongly agree**

| 1 Some people (teachers, classmates, relatives) are there for me when I have problems. | 1 | 2 | 3 | 4 | 5 | 6 | 7 |
| --- | --- | --- | --- | --- | --- | --- | --- |
| 2 There are people (teachers, classmates, relatives) with whom I can share my joys and sorrows. | 1 | 2 | 3 | 4 | 5 | 6 | 7 |
| 3 My family is able to help me in a concrete way. | 1 | 2 | 3 | 4 | 5 | 6 | 7 |
| 4 I can get emotional help and support from my family when I need it. | 1 | 2 | 3 | 4 | 5 | 6 | 7 |
| 5 Some people (teachers, classmates, relatives) are a real source of comfort when I am in trouble. | 1 | 2 | 3 | 4 | 5 | 6 | 7 |
| 6 My friends can be a real help to me | 1 | 2 | 3 | 4 | 5 | 6 | 7 |
| 7 I can rely on my friends when difficulties occur | 1 | 2 | 3 | 4 | 5 | 6 | 7 |
| 8 I can talk about my difficulties with my own family | 1 | 2 | 3 | 4 | 5 | 6 | 7 |
| 9 My friends can share my joys and sorrows with me | 1 | 2 | 3 | 4 | 5 | 6 | 7 |
| 10 There are some people in my life (teachers, classmates, relatives) who care about my feelings. | 1 | 2 | 3 | 4 | 5 | 6 | 7 |
| 11 My family can willingly assist me in making various decisions | 1 | 2 | 3 | 4 | 5 | 6 | 7 |
| 12 I can discuss my problems with my friends | 1 | 2 | 3 | 4 | 5 | 6 | 7 |
